# The Role of Signalling Pathways in Myocardial Fibrosis in Hypertrophic Cardiomyopathy

**DOI:** 10.31083/RCM27152

**Published:** 2025-02-21

**Authors:** Patryk Skórka, Jakub Piotrowski, Estera Bakinowska, Kajetan Kiełbowski, Andrzej Pawlik

**Affiliations:** ^1^Department of Physiology, Pomeranian Medical University, 70-111 Szczecin, Poland

**Keywords:** hypertrophic cardiomyopathy, signalling pathways, transforming growth factor-β1, cardiac fibrosis

## Abstract

Hypertrophic cardiomyopathy (HCM) is the most prevalent hereditary cardiovascular disorder, characterised by left ventricular hypertrophy and cardiac fibrosis. Cardiac fibroblasts, transformed into myofibroblasts, play a crucial role in the development of fibrosis. However, interactions between fibroblasts, cardiomyocytes, and immune cells are considered major mechanisms driving fibrosis progression. While the disease has a strong genetic background, its pathogenetic mechanisms remain complex and not fully understood. Several signalling pathways are implicated in fibrosis development. Among these, transforming growth factor-beta and angiotensin II are frequently studied in the context of cardiac fibrosis. In this review, we summarise the most current evidence on the involvement of signalling pathways in the pathogenesis of HCM. Additionally, we discuss the potential role of monitoring pro-fibrotic molecules in predicting clinical outcomes in patients with HCM.

## 1. Introduction 

**Fig. 1.**
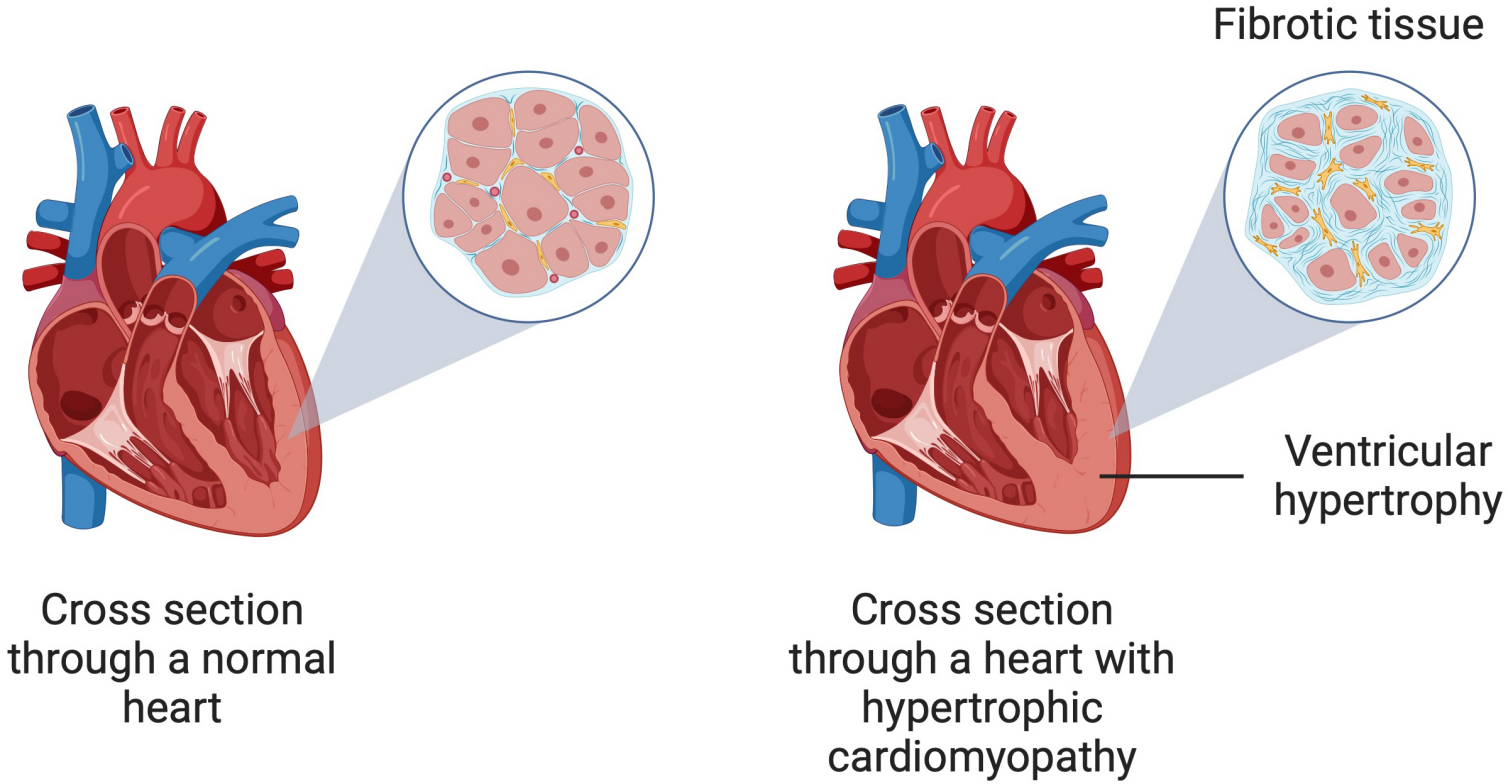
**A schematic illustration of hypertrophic cardiomyopathy showing ventricular hypertrophy and myocardial fibrosis**. Created in BioRender. Kiełbowski, K. (2024) https://BioRender.com/q07s623.

Hypertrophic cardiomyopathy (HCM) is the most common hereditary cardiovascular disease, characterized by left ventricular hypertrophy (LVH) and myocardial fibrosis [1]. It is estimated that HCM affects 0.2% to 0.5% of the general population [2, 3]. One recent study indicate that the prevalence may be higher than previously thought due to underdiagnosis and the variable expression of genetic mutations [4]. HCM is familial in approximately 60% of cases, with first-degree relatives of affected individuals having a 50% probability of inheriting the condition [5]. This condition follows an autosomal dominant inheritance pattern, whereby a single mutation in one of the genes is sufficient to cause HCM [6]. The disease may manifest at any age, but the clinical symptoms typically emerge during adolescence or early adulthood. In some patients, however, disease progression may be delayed [5]. HCM is associated with an elevated risk of sudden cardiac death (SCD), particularly among young athletes, where it is one of the leading causes of SCD [7]. Consequently, HCM represents a significant contributor to hospitalizations and is a risk factor for heart failure in patients with advanced stages of the disease. Despite recent advances in diagnostic techniques and therapeutic modalities, a significant number of cases remain undiagnosed, thereby complicating the development of effective preventive and therapeutic strategies [8]. The underlying pathogenesis involves mutations in sarcomeric proteins, such as encoding β cardiac myosin heavy chain (MYH7) or encoding cardiac myosin binding protein-C (MYBPC3), which are the most common (up to 70%) and are essential for cardiomyocyte contractility and structural integrity. Furthermore, mutations in non-sarcomeric proteins, including phospholamban (PLN) and filamin C (FLNC), have been identified as significant contributors to HCM [9, 10]. While mutations affecting the sarcomere disrupt cardiomyocyte contractile function [11], mutations occurring outside the sarcomere impact cellular stability and calcium handling, thereby contributing to hypertrophic remodeling and increased arrhythmic risk [10, 12]. Although HCM is primarily considered a disease of the cardiomyocytes, growing evidence suggests that profibrotic signaling is induced early in the disease process [13] (Fig. 1). The extent of fibrosis is closely correlated with disease progression and adverse clinical outcomes in HCM, thereby underscoring fibrosis as an important therapeutic target [14, 15]. This fibrotic remodeling, which arises from both genetic and biomechanical factors, remains a central focus of research efforts aimed at improving prognosis and quality of life in HCM patients. Myocardial fibrosis has a profound impact on the clinical condition of the patient. For instance, fibrosis is associated with the occurrence of arrhythmias. Fibrotic tissue impairs electrical coupling between cardiac cells [16]. Moreover, cardiomyocytes can electrically couple with fibroblasts, which affects conductivity if scar tissue is present in the heart [17]. In this review, we will discuss the current evidence on the genetic effectors and molecular signaling pathways that are involved in the development of myocardial fibrosis in HCM. Proper understanding of these pathways could lead to the development of targeted therapeutics that would suppress or even reverse the process of fibrosis.

## 2. Pathophysiology and Current Treatment Strategies of HCM

Myocardial fibrosis is a complex and multifactorial process, which can develop after primary inflammatory processes, including cardiac sarcoidosis, acute myocarditis or genetically determined cardiomyopathies [18, 19, 20]. Furthermore, immune dysregulation and chronic inflammation, cardiac damage due to myocardial infarction, or pressure overload are also involved in the process of cardiac fibrosis development [21, 22, 23]. It leads to negative remodeling of the interstitial structure of the myocardium through excessive deposition of extracellular matrix (ECM) proteins in the cardiac interstitia. Collagen is synthesized most intensively, leading to an increased percentage of collagen fibers throughout the myocardial tissue [24, 25]. Therefore, the ECM plays a key role in regulating cardiac function [21]. Two types of fibrosis predominate in HCM—interstitial-perimyocytic and scar-like (replacement) fibrosis. It is estimated that up to one-third of the myocardium in HCM is covered by fibrosis, leading to asymmetric left ventricular thickening, reduced compliance, and progressive myocardial fibrosis. Furthermore, a degree of left ventricular fibrosis exceeding 20% is considered a high-risk condition associated with significant diastolic dysfunction [26].

Cardiac fibroblasts play a central role in the fibrosis process because they mediate intercellular communication between cardiomyocytes, inflammatory cells, and endothelial cells [27]. The phenotypic change of fibroblasts to myofibroblasts leads to a reduced rate of ECM degradation and increased collagen synthesis [28]. Additionally, the ECM influences cells such as myocytes and macrophages by regulating mechanical tension and controlling the availability of growth factors and matrix proteins [29]. Fibroblast activation occurs through communication between macrophages, fibroblasts, and cardiomyocytes in cardiac tissue, with chemokines and inflammatory cytokines playing a role in inducing this phenotypic change.

The primary factors promoting fibroblast transition include endothelin-1, angiotensin II (Ang II), platelet-derived growth factor (PDGF), fibroblast growth factor, interleukin (IL)-6, IL-4, IL-1, and transforming growth factor-β (TGF-β). Mouse models have demonstrated that reduced IL-1β levels result in increased TGF-β, which inhibits the induction of myocardial fibrosis, although the exact mechanism remains under investigation [30]. TGF-β isoforms play a key role in the activation of fibroblasts [29]. In the early stages after injury, TGF-β activation has a predominantly anti-inflammatory effect. However, prolonged and excessive activation of this signalling pathway in fibroblasts and mechanically stressed cardiomyocytes leads to myocardial dysfunction [31].

Genes encoding key proteins likely involved in this process are noteworthy. For instance, the type I/II collagen-binding prolargin encoded by *PRELP* and the *COL22A1* gene, which encodes the alpha chain of collagen XXII, expressed in tendon-muscle junctions, are implicated [32]. These mechanisms, involving mitochondrial and cellular stress, contribute to the hypertrophic response in the heart. Although the initiating signalling pathways vary, they converge on common pathways that alter cell metabolism and result in HCM [33].

Late gadolinium enhancement (LGE) and extracellular volume (ECV) may be key indicators in the diagnosis of HCM. Although both indices are highly effective in the assessment of sudden cardiac death, it is LGE that has a stronger association with the risk of non-sustained ventricular tachycardia (NSVT) and diastolic dysfunction [34]. The determined receiver operating characteristic curve (ROC) curve for the extent of LGE in specific left ventricular segments obtained a high area under curve (AUC) value of 0.861 [35]. An even more reliable and repeated instrument in assessing risk classification in HCM based on variability and extent of cardiac scarring is LGE entropy [36]. Similar to the LGE entropy, LGE rate may discover an important role in the surveillance and monitoring of HCM patients [37]. Therefore, a thorough understanding of the molecular mechanisms regulating LGE in patients with HCM, may contribute to the stratification of protein biomarkers by proteomic profiling [38].

**Fig. 2.**
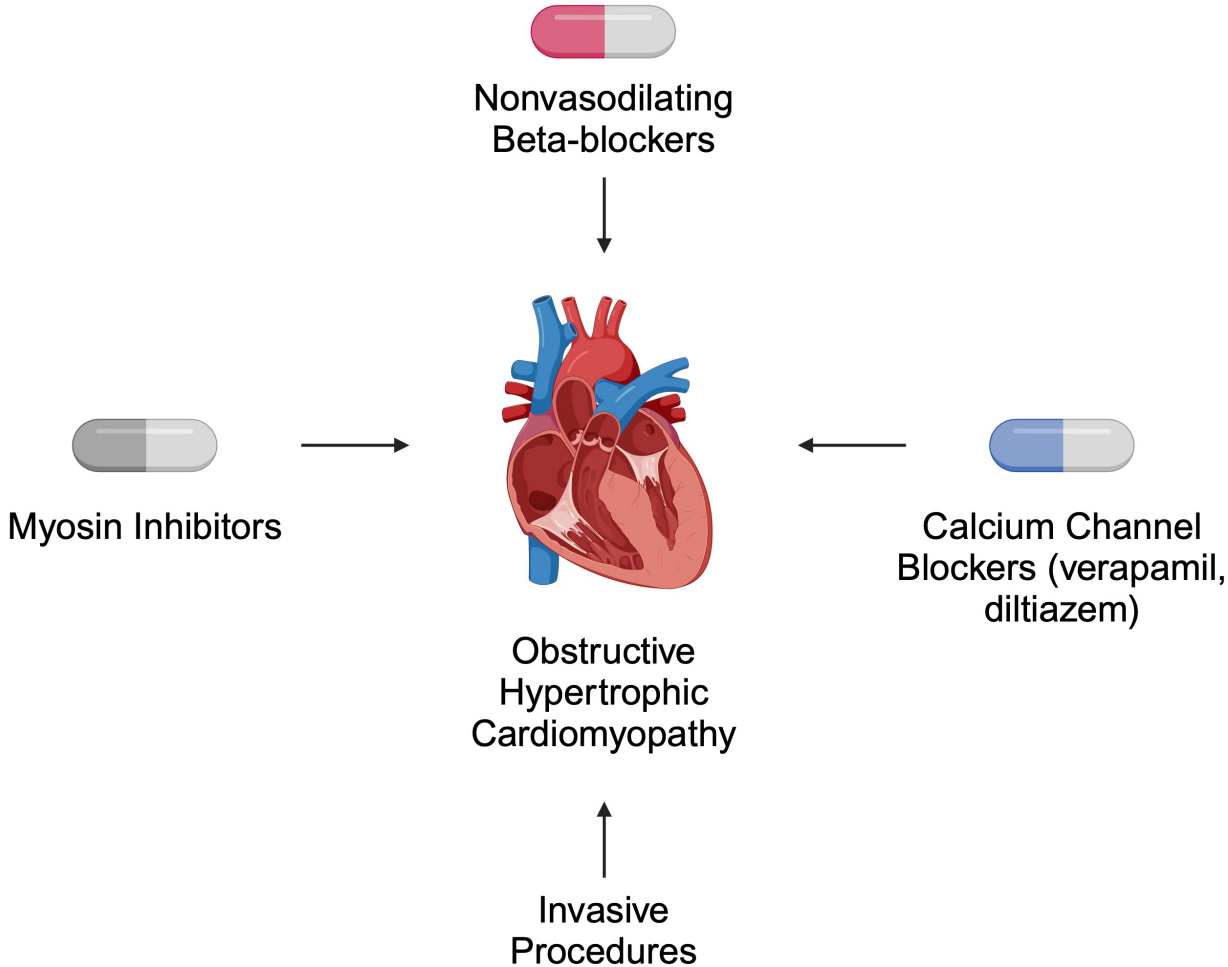
**Selected recommended treatment strategies for patients with obstructive hypertrophic cardiomyopathy**. Created in BioRender. Kiełbowski, K. (2024) https://BioRender.com/a56h896.

Both pharmacological and invasive treatment strategies are recommended for patients with HCM. In obstructive disease, the primary role of pharmacotherapy is symptom relief [39]. According to the 2024 American Heart Association (AHA)/American College of Cardiology (ACC)/American Medical Society for Sports Medicine (AMSSM)/Heart Rhythm Society (HRS)/Pediatric and Congenital Electrophysiology Society (PACES)/Society of Cardiovascular Magnetic Resonance (SCMR) guidelines, non-vasodilating beta-blockers are suggested as first-line therapy. Verapamil and diltiazem are alternatives for patients who cannot tolerate beta-blockers. For non-responders, myosin inhibitors such as mavacamten are recommended. Invasive approaches, such as septal reduction therapy, are offered to patients who do not respond to pharmacotherapy [39] (Fig. 2).

## 3. Genetic Basis of HCM and Its Impact on Fibrosis 

HCM is an example of single-gene disorder, characterized by an autosomal dominant inheritance pattern. Genetic mutations disrupt the structure and functionality of the myocardium, exerting a profound influence on the fibrotic processes that define disease progression [15]. The primary genetic contributors to HCM are mutations in sarcomere proteins, particularly those in the *MYH7* and *MYBPC3* genes, which are the most frequently implicated genes across global HCM populations [40]. These genes encode β-myosin heavy chain and myosin-binding protein C, respectively, both of which are essential for the contractile function and structural stability of cardiomyocytes [41]. It has been observed by several authors that patients with HCM and sarcomere mutations exhibit more fibrosis than those who tested negative for genetic mutations [42, 43]. *MYH7* mutations have the effect of disrupting normal sarcomere function, thereby impairing the force generation necessary for effective heart contractions. This mechanical disruption results in an increased mechanical load on the myocardium, which in turn activates profibrotic signaling pathways, including TGF-β and Ang II, that promote fibroblast activation and excessive collagen deposition within the ECM [44]. This fibrotic process not only increases myocardial stiffness but also disrupts normal electrical conduction, thereby contributing to arrhythmic risks in patients with HCM [45]. Mutations in *MYBPC3* similarly contribute to fibrosis through a different mechanism. The disruption of sarcomere organization and induction of cellular stress, particularly oxidative stress, that occurs as a result of *MYBPC3* haploinsufficiency has been identified as a known promoter of fibrotic remodeling. This process results in the secretion of cytokines that enhance fibroblast proliferation, thereby increasing ECM deposition and further contributing to myocardial rigidity and impaired relaxation [46, 47]. In summary, the aforementioned sarcomeric protein mutations result in a cycle of cellular stress and fibrotic remodeling, which has been linked to adverse clinical outcomes in HCM patients.

Non-sarcomeric genetic mutations play a substantial role in the fibrotic processes observed in HCM, influencing cellular stability, calcium homeostasis, and overall myocardial function [48]. Mutations in the *PLN* gene affect calcium cycling by impairing the reuptake of calcium into the sarcoplasmic reticulum, which results in elevated cytoplasmic calcium concentrations [49, 50]. This dysregulation of calcium handling not only disrupts cardiomyocyte relaxation but also activates profibrotic pathways [51]. An increase in cytoplasmic calcium is associated with the activation of the TGF-β pathway [52], which enhances fibroblast differentiation and collagen production, resulting in a stiffer and less compliant myocardium. The role of *FLNC* mutations serves to underscore the significance of non-sarcomeric factors in HCM fibrosis [53, 54, 55]. *FLNC* plays a pivotal role in maintaining cytoskeletal integrity. Mutations in *FLNC*, therefore, compromise this structural framework, creating instability within the cardiomyocytes [56]. This instability results in increased mechanical stress, which activates integrin-related signaling pathways that stimulate fibroblast activation and collagen synthesis [57]. Furthermore, studies have demonstrated that *FLNC *mutations interact with ECM components, thereby reinforcing fibrosis by sustaining collagen production and enhancing myocardial stiffness [58, 59]. This increased fibrotic remodeling due to non-sarcomeric mutations highlights the complex genetic mechanisms contributing to fibrosis in HCM and identifies potential therapeutic targets for modulating fibrosis in affected individuals.

## 4. Cellular Mechanisms of Fibrosis in HCM

Cardiac fibrosis is a pathological process characterised by the excessive deposition of ECM proteins in response to pathophysiological stimuli, leading to scarring of heart tissue. Patients with HCM experience significant levels of cardiac fibrosis, which can result in diastolic dysfunction [26]. One of the primary drivers of fibrosis is fibroblast activation. Fibroblasts transform into myofibroblasts, producing an excess of ECM. The interaction between cardiomyocytes and fibroblasts, such as through the TGF-β pathway, promotes progressive fibrosis and reduces the flexibility and contractile function of the heart [60, 61]. Concurrently, cardiomyocytes respond to the stress of cardiac hypertrophy by releasing pro-inflammatory cytokines that influence fibroblast activity [62]. Understanding the regulatory processes governing fibroblast activation in HCM could help mitigate cardiac fibrosis and support the development of effective medical interventions to prevent adverse outcomes in patients [63].

The cellular mechanisms of fibrosis in HCM involve intricate interactions between cardiomyocytes, fibroblasts, and immune system cells. Endothelial-to-mesenchymal transition (EndoMT) is a process in which endothelial cells transform into mesenchymal cells, promoting fibrosis [64]. In HCM, EndoMT contributes to increased numbers of fibroblasts and myofibroblasts within cardiac tissue. It is suggested that factors such as TGF-β and hypoxia induce EndoMT, leading to elevated ECM production and advancing fibrosis [65]. Furthermore, cells undergoing EndoMT exhibit altered gene expression, which affects their function and interactions with other cardiac cells [66].

Cells of the immune system, such as macrophages and lymphocytes, also play an important role in fibrosis processes in HCM [67]. In patients with HCM and acute clinical worsening, myocardial biopsy often demonstrates inflammatory infiltration that is associated with necrosis of myocytes [22]. Pro-inflammatory cytokines such as tumour necrosis factor-α (TNF-α), IL-1β, and IL-6 are markedly elevated in various myocardial pathological conditions associated with fibrosis. A clinical study has shown a correlation between elevated levels of these cytokines and fibrosis-related endpoints [68]. TNF-α facilitates fibrogenic activation of immune cell populations within injured myocardium. In mice overexpressing TNF-α, the fibrogenic actions of TNF-α in the myocardium have been partly attributed to the expansion of cardiac mast cells [69]. The precise role of neutrophils in HCM remains to be determined. However, it is established that these cells release pro-inflammatory factors and enzymes, such as myeloperoxidase and acid phosphatase, which can directly damage the myocardium. This leads to irregular thickening of fibers and contributes to ECM degradation and fibroblast activation [70]. The prolonged presence of neutrophils can result in chronic inflammation, which subsequently facilitates the development of HCM. Understanding the cellular mechanisms leading to fibrosis in hypertrophic cardiomyopathy will allow the development of effective therapeutic strategies. Interactions between cardiomyocytes and fibroblasts, EndoMT and the role of immune cells are important areas of research that can contribute to the understanding and treatment of this complex disease.

## 5. Key Signaling Pathways in HCM-related Fibrosis

### 5.1 TGF-β Signaling Pathway

When discussing tissue fibrosis and signaling pathways, it is crucial to focus on TGF-β and its downstream elements. This molecule signals through TGF-β receptors, promoting intracellular canonical signalling via Smads. These proteins mediate the downstream effects of TGF-β. Upon ligand binding, Sma and Mad related protein (SMAD)3 and SMAD4 are phosphorylated and interact with SMAD4. The resulting complex enters the nucleus to induce gene expression. SMAD7 acts as a negative regulator of this pathway [71]. By acting on fibroblasts, TGF-β stimulates the expression of collagens and inhibitors of matrix metalloproteinases, thereby increasing ECM stability. Additionally, this pro-fibrotic molecule affects immune cells, contributing to fibrosis formation. For instance, TGF-β enhances macrophage presence and their pro-fibrotic responses [72].

Several studies have investigated the relationship between TGF-β and cardiac hypertrophy. The use of TGF-β neutralising antibodies in mice treated with cyclosporin A to induce HCM has been associated with significantly reduced ventricular thickness, a lower presence of fibrosis, and decreased rates of non-myocyte proliferation. These findings confirm the important role of TGF-β in the pathogenesis of HCM [73]. Immunohistochemical analyses of the hearts of larger animals with HCM also show a greater presence of TGF-β [74]. More recently, a large-scale proteomic study demonstrated that the TGF-β protein is upregulated in the blood of patients with HCM [75]. Furthermore, the expression of SMAD2 and SMAD3, which are considered as downstream pro-fibrotic elements of TGF-β, are increased in cardiac samples of patients with hypertrophic obstructive cardiomyopathy [76].

Huang *et al*. [77] recently examined TGF-β-related genes in single-cell RNA sequencing and transcriptome datasets of heart failure and cardiac hypertrophy. The analyses revealed that TGF-β pathways are stimulated in cells affected by heart failure. Furthermore, researchers analysed differentially expressed genes in the dataset of cardiac hypertrophy, identifying aberrant expression of several in this cohort. Six hub genes (*ADAMTS2*, *TANC2*, *MRC2*, *DYNLL1*, *OTUD1*, and *EGR1*) were identified as potential therapeutic targets for heart failure and cardiac hypertrophy. Notably, some of these genes were significantly associated with cardiac fibrosis. For instance, early growth response 1 (EGR1) is a pro-fibrotic molecule implicated in the fibrosis of various tissues [78, 79]. TGF-β can stimulate the expression of EGR1 through a SMAD-independent mechanism mediated by the mitogen-activated protein kinase (MAPK) pathway [80]. Importantly, EGR1 has been shown to enhance the expression of fibrosis-associated genes, including *Tgf-β3*, *Pdgf-a*, *Pdgf-b*, and *Col1α2*. In cardiac fibrosis, EGR1 mediates pro-fibrotic alterations as a downstream element of Ang II [81]. EGR1 appears to be a significant fibrotic stimulator in the heart. A recent study by Laggner *et al*. [82] confirmed that it is upregulated in hearts with right ventricular hypertrophy due to pulmonary hypertension.

Apart from EGR1, TGF-β is associated with other molecules closely related to cardiac fibrosis. Lysine oxidase (LOX) is an enzyme that catalyses the oxidative deamination of lysine and hydroxylysine residues in collagen. This process initiates inter- and intramolecular covalent cross-linking, altering collagen stiffness. TGF-β upregulates the expression of LOX in cardiac fibrosis [83], thereby influencing cardiac cross-linking. In patients with obstructive HCM who underwent surgical myomectomy, collagen levels were significantly higher than in a control group composed of accident victims. Additionally, tissue samples from patients with HCM showed increased LOX expression, which correlated with the presence of collagen fibres [84].

Recently, TGF-β has been linked to the activity of endothelin-1, a molecule associated with pathological changes in HCM [85, 86]. In rat cardiomyocytes, endothelin-1 promotes the expression of *TGF-β*1-3, along with TGF-β protein production and secretion. It may also affect cardiac fibroblasts, inducing pro-fibrotic changes. In the same study, Liu *et al*. [87] analysed data from human samples and found that only *TGF-β2* and *TGF-β3* genes were upregulated in human tissues. Furthermore, the researchers observed increased expression of the anti-fibrotic gene *SMAD7*, a negative regulator of TGF-β signalling. Hypothetically, this upregulation could represent a compensatory mechanism aimed at mitigating fibrotic alterations in cardiac tissue. In another study, the expression of SMAD7 was reduced in patients with obstructive HCM [76].

Recently, significant attention has been directed toward regulatory mechanisms that mediate gene expression. These epigenetic mechanisms often involve non-coding RNA, such as microRNA (miRNA). These small molecules, typically about 20 nucleotides in length, bind to their target mRNA to suppress translation. The importance of miRNAs was highlighted in 2024 when the Nobel Prize in Medicine and Physiology was awarded to the discoverers of these molecules [88].

More than 10 years ago, Bagnall *et al*. [89] analyzed the miRNA profile in a double mutant mouse HCM model. Among their findings, the authors observed that microRNA- 1 (miR-1) expression was reduced to a pre-disease state. This finding aligns with more recent research. Specifically, TGF-β receptor 1 was identified as a target of miR-1-5p, and reduced expression of this miRNA was also found in animal models of right ventricular hypertrophy [90]. The downregulation of miR-1 could result in an increased presence of TGF-β receptors, thus amplifying the pro-fibrotic processes observed in hypertrophy.

Monitoring TGF-β could potentially serve as a tool for evaluating the prognosis of patients with HCM. In a study by Shimada *et al*. [91], the researchers demonstrated that upregulated TGF-β pathways were associated with major adverse cardiovascular events (MACEs) in patients with HCM. In another study, researchers employed proteomic analyses to develop a diagnostic panel for distinguishing patients with HCM who had a history of MACEs. In total, 571 proteins were differentially regulated in HCM patients with and without a history of MACEs. A diagnostic model comprising 29 proteins, including TGF-β, achieved an area under the curve of 0.82 [92]. Additionally, monitoring the circulating TGF-β was suggested to indicate the possible occurrence of postoperative atrial fibrillation in patients with HCM undergoing surgical treatment [93].

### 5.2 Renin–Angiotensin–Aldosteron System (RAAS)

The effect of Ang II on cardiac hypertrophy depends on the activation of specific receptors. Activation of the pro-hypertrophic Ang type 1 receptor stimulates phosphorylase C, promotes protein kinase C, and mobilises Ca^2+^ ions. This cascade activates nicotinamide adenine dinucleotide phosphate (NADPH) oxidase and alters cardiomyocyte metabolism, ultimately leading to pathological cardiac hypertrophy [94]. Interestingly, Ang II enhances the expression of connective tissue growth factor, an ECM protein, while downregulating epidermal growth factor receptor expression. This regulation is mediated by extracellular signal-regulated kinase 1/2 (ERK1/2), a member of the MAPK pathway [95]. An important mechanism has been observed in a mouse model, where Ang II and phenylephrine infusion resulted in greater upregulation of insulin-like growth factor-1 (IGF-1) receptor expression in myofibroblasts than in cardiomyocytes. The study also suggested that IGF-1 attenuates interstitial myocardial fibrosis by downregulating the expression of rho-associated coiled-coil containing kinase (ROCK)2-mediated α-smooth muscle actin (α-SMA) and Akt signalling pathways [96].

Another mechanism contributing to myocardial fibrosis involves the effect of Ang II on the upregulation of wingless-type MMTV integration site family, member 3a (WNT3a) in cardiomyocytes. This promotes the paracrine transformation of fibroblasts, enhancing fibrosis through increased expression of α-SMA and fibronectin [97]. The Ang II/WNT/β-catenin regulatory pathway, which is influenced by protein arginine methyltransferase 7 (PRMT7), is particularly important. Reduced PRMT7 expression was observed in cardiomyocytes subjected to continuous Ang II infusion in an animal model. The study suggested that PRMT7-mediated methylation of axin stabilises axin and β-catenin, leading to β-catenin degradation and inhibiting Ang II-induced negative cardiac remodelling [98].

The literature demonstrates conflicting results on the efficacy of RAAS inhibition in reducing cardiac fibrosis in patients with HCM [99]. Recently, the phase 2b clinical trial investigated the use of valsartan (AngII receptor blocker) showed improved cardiac functionality and structure in patients with early stage HCM [100]. However, according to the VANISH trial, valsartan was not effective in patients with subclinical HCM [101].

### 5.3 JAK/STAT Pathway

The interaction of a ligand with its corresponding receptor activates the Janus kinase (JAK)/signal transducers and activators of transcription 3 (STAT3) signalling pathway. This leads to autophosphorylation of the JAK kinase, which is bound to the receptor, by phosphorylating its tyrosine residue. STAT3 is subsequently phosphorylated, causing it to dissociate and form a dimer. These STAT3 dimers regulate transcription by binding to promoters in the cell nucleus. Because this pathway is activated by factors promoting fibroblast activation—such as endothelin-1, Ang II, vascular endothelial growth factor (VEGF), PDGF, TGF-β1, and IL-6—it plays a critical role in cardiac fibrosis and may contribute significantly to HCM pathology [102]. High levels of IL-6 during cardiopulmonary bypass likely contribute to myocardial fibrosis through JAK/STAT activation [103].

Interestingly, the peptide hormone Elabela inhibited myocardial fibrosis in mouse models by suppressing the IL-6/STAT3 signalling pathway and activating the cystine–glutamate antiporter xCT/glutathione peroxidase pathway [104]. Obesity and diet are also significant factors influencing HCM progression. Animal studies have shown that a high-fat, corn oil-rich diet that induces a type 2 diabetes phenotype promotes left ventricular collagen synthesis. This occurs through increased IL-6 and reactive oxygen species synthesis, activating the JAK1/STAT3/Ang II/TGF-β1/SMAD3 pathway [105].

Furthermore, adipose tissue in obese individuals leads to elevated levels of the adipokine resistin, which promotes fibroblast differentiation by activating the JAK2/STAT3 and c-Jun N-terminal kinase (JNK-cJun) pathways. This, in turn, drives the expression of numerous ECM proteins, contributing to chronic cardiac fibrosis, particularly in overweight and diabetic individuals [106]. Aerobic training over a 28-day period has been shown to inhibit negative myocardial remodelling via miR-574-3p in animal models, which suppresses IL-6 [107]. miR-326, inhibits myocardial hypertrophy by downregulating the JAK/STAT and MAPK signalling pathways. These miRNA molecules may have significant roles in HCM progression and represent potential therapeutic targets [108]. Women with HCM experience significantly greater age-related deterioration in heart function after menopause than men of the same age group [109]. Protein arginine methyltransferase 7 (PRMT7) is an important factor in this pathology. Reduced PREMT7 expression in the cardiomyocytes of postmenopausal women diminishes the alleviation of inflammation and oxidative stress associated with menopause. PRMT7, through STAT3 activation, induces the expression of SOCS3, a direct inhibitor of the JAK/STAT pathway [110]. STAT3 regulates the expression of Collagen-IV (Col-IV) which is linked to interstitial fibrosis. STAT3/Col-IV expression has been shown to be different between HCM patients with different phenotypes of fibrosis [111]. Table 1 (Ref. [73, 76, 84, 99, 100, 101, 111]) presents a summary of the potential involvement of TGF-β, RAAS and JAK/STAT in HCM and cardiac fibrosis.

**Table 1.** **Selected mechanisms involving TGF-β, RAAS and JAK/STAT3 pathways in HCM and cardiac fibrosis**.

Pathway	Relationship with hypertrophic cardiomyopathy and cardiac fibrosis	References
TGF-β	TGF-β neutralising antibodies suppress HCM progression and fibrosis in mice.	[73, 76, 84]
	TGF-β stimulates LOX, which influences collagen stiffness and is correlated with cardiac fibrosis.	
	Increased expression of SMAD2 and SMAD3 (pro-fibrotic signalling elements of the TGF pathway) was found in patients with obstructive HCM.	
RAAS	The use of valsartan provided beneficial outcomes in patients with early HCM. However, conflicting results were published regarding the inhibition of RAAS and cardiac fibrosis.	[99, 100, 101]
JAK/STAT3	Analysis of STAT3 could be used to analyze fibrosis phenotypes in patients.	[111]

TGF-β, transforming growth factor β; LOX, lysine oxidase; RAAS, renin angiotensin aldosterone system; JAK/STAT3, Janus kinase 2/signal transducer and activator of transcription 3; HCM, hypertrophic cardiomyopathy; SMAD, mothers against decapentaplegic homolog.

### 5.4 WNT/β-catenin Pathway

The WNT/β-catenin pathway is a central signalling pathway involved in cardiac fibrosis through the activation of cardiac fibroblasts [112]. While this pathway plays an essential role in the reparative phase following myocardial infarction, prolonged profibrotic effects can lead to negative outcomes, such as HCM progression [113]. The importance of this pathway was demonstrated in a rat study using a WNT/β-catenin/c-Myc/cyclin D1 pathway antagonist, which reduced the expression of these proteins and decreased levels of collagen-I/-III (Col-I/-III), vimentin, and α-SMA. Additionally, inhibition of negative cardiac remodelling was observed [114].

WNT3a and WNT5a are key ligands promoting this pathway, although they act through different mechanisms. WNT3a facilitates the nuclear translocation of β-catenin by inhibiting Glycogen Synthase Kinase 3β (GSK3β), allowing the activation of T-cell factor (TCF)/lymphoid enhancer factor (LEF) transcription and genes such as *AXIN2* and *TCF7*. By contrast, WNT5a does not inhibit GSK3β; instead, it leads to IL-6 production and activates the JNK, ERK1/2, and activator protein 1 (AP1) pathways, which are unaffected by WNT3a [115].

The profibrotic response can be further enhanced by the synergistic effects of WNT3a and TGF-β through the promotion of IL-11 synthesis. Additionally, TGF-β1 enhances WNT3a expression by regulating the ALK5/Smad2/3 axis, which increases c-Myc expression—a process also promoted by WNT/β-catenin [116]. Interestingly, WNT5a can inhibit TGF-β-induced Col- I synthesis.

In mice with myocardial infarction, collagen synthesis was inhibited by circNSD1 knockdown. CircNSD1 acts as a sponge for miRNA-429-3p, and its knockdown reduces sulfatase1(SULF1) expression and WNT/β-catenin activation, thereby inhibiting collagen synthesis in myofibroblasts [117]. Another molecule of interest is circular RNA ArfGAP with RhoGAP domain, ankyrin repeat and PH domain 1 (circARAP1), which promotes the miR-379-5p/Krüppel-like factor 9 (KLF9)/WNT/β-catenin axis [118]. The use of inhibitors such as pyrrolidine dithiocarbamate in rat models has shown decreased levels of TNF-α, reduced pro-inflammatory cytokines, and inhibition of Wnt/β-catenin/GSK3β and nuclear Factor kappa-light-chain-enhancer of activated B cells (NF-κB) pathways [119]. A substance with high clinical potential is the porcupine inhibitor CGX1321. In mouse and rat models, CGX1321 significantly reduced fibrosis and myocardial hypertrophy while improving right ventricular function by reducing the expression of Fos like antigen 1 (FOSL1), WNT3a, and WNT5a, thereby inhibiting both canonical and non-canonical pathways [120, 121].

Doxorubicin has been shown to activate the WNT/β-catenin pathway, along with the RAAS components, including renin, angiotensin-converting enzyme (ACE), and angiotensin II type 1 receptor (AT1). This activity can be mitigated by infliximab [122].

A potential protein enhancing the production of fibrosis-associated compounds, such as α-SMA, Col-I, and fibronectin, is neuroepithelial cell-transforming gene 1 protein (NET1). In a mouse model, upregulated NET1 formed a complex with GSK3β phosphatase, enabling the creation of the NET1-GSK3β-β-catenin complex. This complex promotes β-catenin nuclear transport, preventing ubiquitin-dependent protein degradation [123].

Finally, inhibition of Col-I/III and α-SMA expression in a mouse model was mediated by downregulated dedicator of cytokinesis 2 (DOCK2), which led to suppression of the WNT/β-catenin pathway [124].

### 5.5 MAPK Pathway

Molecules belonging to the MAPK family play a significant role in negative cardiac remodelling. These include kinases such as ERK1/2, p38MAPK, and JNK1/2 [125]. Suppression of ERK in a mouse model of cardiomyopathy inhibited myocardial fibrosis [126]. One mechanism for the inhibition of cardiac fibroblasts involves SO_2_-induced sulphenylation of ERK1/2 [127]. Additionally, the use of ERK1/2 inhibitors may counteract the profibrotic effects elicited by the TNF family member CD137 [128]. Myocardial fibrosis can also be mitigated through inhibition of the ERK1/2-matrix metalloproteinase-9 (MMP- 9) axis via activation of the G protein-coupled oestrogen receptor 30 [129].

The SerpinE2 protein exhibits antagonistic effects, interacting with membrane proteins such as low-density lipoprotein receptor-related protein 1 (LPR1) and urokinase-type plasminogen activator receptor (uPAR) to activate ERK1/2 and β-catenin, thereby increasing collagen production [130]. Activation of the ERK1 pathway can also occur via cytokine receptor-like factor 1, whose elevated expression was observed in TGF-β1-stimulated human cardiac fibroblasts [131].

The p38MAPK plays a crucial role in cardiac fibrosis and hypertrophy. Inhibition of this kinase reduces cardiac hypertrophy and preserves collagen synthesis in neonatal rat cardiac myocytes and IGF-1-induced fibroblasts [132]. In septic mice, α1-adrenergic receptor (α1-AR) agonists in the cardiac endothelium inhibit cardiac remodelling. Activation of the α1-AR receptor promotes protein kinase C, leading to ERK1/2 activation, while inhibiting the p38MAPK pathway, which is activated through toll-like receptor 4 by lipopolysaccharides [133].

Interestingly, p38MAPK activation leads to a decrease in Cx43, a protein required for normal myocardial function, disrupting the balance between microtubule depolymerisation and polymerisation in the myocardium [134]. IL-17 regulates p38MAPK, promoting negative cardiac remodelling by upregulating C-C motif ligand 2 (CCL2) and activating AP-1 via the adaptor protein 1 (Act1)/TNF receptor-associated factor 6 (TRAF6)/p38MAPK pathway [135]. JNK1/2 also contributes to the synthesis of profibrotic factors through the transforming growth factor beta-activated kinase 1-p38 (TAK1-p38)/JNK1/2 regulatory axis. Ubiquitin-specific protease 19 negatively affects this axis by inhibiting the transition of fibroblasts to myofibroblasts [136]. Similarly, zinc finger protein zinc finger and BTB domain containing 20 (ZBTB20) may inhibit fibrosis after myocardial infarction by targeting the TNF-α/apoptosis signal-regulating kinase 1 (ASK1)/JNK1/2 pathway, exerting an anti-apoptotic effect [137].

In HCM, Kyoto Encyclopedia of Genes and Genomes (KEGG) enrichment analysis demonstrates significant dysregulation of the MAPK pathway [138]. Furthermore, dysregulation of MAPK signalling is associated with the worsening of heart failure [139].

## 6. Mitochondrial Dysfunction and HCM

In the previous sections, we have focused on the genetic background of HCM pathogenesis and the potential involvement of signaling pathways in hypertrophy and cardiac fibrosis. Recently, there is growing attention towards mitochondrial dysfunction as a pathogenic element in the occurrence of HCM. Analyses of the HCM cardiac samples demonstrate impaired energy metabolism. Diseased hearts show signs of disrupted fatty acid metabolism, with reduced expression of enzymes involved in metabolism and transport of acylcarnitines. Moreover, the state of HCM is associated with impaired structure of mitochondria and their ability to perform oxidative phosphorylation, together with reduced levels of ATP, thus demonstrating energy deprivation [33]. In addition, HCM hearts show alterations in the expression of enzymes involved in the mitochondrial transport of Ca^2+^, which is also involved in adenosine triphosphate (ATP) production [140]. Consequently, disturbances in mitochondrial functionality are considered to be involved in HCM. Perhaps, mitochondrial abnormalities are also linked with the processes of cardiac fibrosis. Tian *et al*. [141] showed that stimulation of cardiac fibroblasts with TGF-β also affects fatty acid oxidation and changes in the expression of enzymes involved in the metabolism of acylcarnitines. Moreover, the authors observed a reduced expression of voltage-dependent anion-selective channel 1 (VDAC-1), a mitochondrial protein that forms a complex with CPT1a mediating acylcarnitines formation, which is thought to also contribute to fatty acid transport. Overexpression of VDAC-1 was found to suppress cardiac fibrosis development [141]. In a recent study, Qian and colleagues [142] showed that using isoflavone compound counteracts mitochondrial dysfunction and alleviates cardiac fibrosis. Thus, improving mitochondrial abnormalities could suppress the mechanisms involved in the pathogenesis of HCM and cardiac fibrosis.

## 7. Modern and Future Therapies in HCM

As previously mentioned, either pharmacological therapy or invasive procedures can be performed in patients with HCM. Pharmacological treatment represents an initial approach in these patients. Recent investigations focus on studying a relatively novel class of therapeutics – myosin inhibitors. An animal study demonstrated mavacamten to suppress cardiac contractility and reduce left ventricle wall thickness. Importantly, if administered early, the drug significantly reduced fibrotic changes in the cardiac tissues. However, administration of the therapeutic in advanced hypertrophy did not reduce fibrotic lesions [143]. Thus, by modulating fibrotic mechanisms, the drug can reduce the risk of developing arrhythmias [16] and further deteriorating heart functionality. The EXPLORER-HCM phase 3 clinical trial proved the efficacy of mavacamten in obstructive HCM, as the drug improved left ventricular outflow tract obstruction and NYHA class, compared to placebo [144]. In 2022, mavacamten was approved by the Food and Drug Administration (FDA) to treat patients with obstructive HCM [145]. A recently published meta-analysis that analyzed 3 randomized controlled trials comparing mavacamten and placebo supports the benefits of myosin inhibition, but warrants further research regarding the issue of treatment-emergent adverse events [146]. Aficamten, a next-generation myosin inhibitor [147], is currently being investigated in patients with HCM. The most recent clinical trials demonstrated the beneficial effects of aficamten in patients with obstructive [148, 149] and nonobstructive HCM [150].

Induced pluripotent stem cells (iPSCs) offer an interesting insight into the pathophysiology of cardiac diseases and potential therapeutic methods. Fibroblasts can be obtained and switched towards iPSCs, which then can be differentiated into cardiomyocytes or other cellular lineages. We have discussed the potential benefits of iPSCs in myocardial infarction in a previous paper [151]. This method is exciting for studying the pathogenesis of diseases, as it is possible to use patient-derived iPSCs and differentiate them towards cardiomyocytes. For instance, Shiba *et al*. [152] generated iPSCs using peripheral blood mononuclear cells of patients with a *DSG2* mutation, which was associated with cardiomyopathy. Researchers then introduced the proper gene to the iPSCs through the adeno-associated viruses. Gene replacement improved the contraction force of the three-dimensional (3D) self-organized tissue rings, thus demonstrating enormous potential in iPSCs. iPSCs are also being used to study cardiac hypertrophy. Rosales and Lizcano [153] studied iPSC-derived cardiomyocytes and identified histone demethylase JMJD2A as a probable enzyme involved in the development of hypertrophy. In the context of this review, iPSC models could be implemented to study signaling pathways or molecules implemented in the development of HCM or HCM-associated cardiac fibrosis.

## 8. Conclusions

HCM is a disease with a complex pathogenesis, involving a genetic background and the activity of several signalling pathways. Aberrant activity of these cascades is associated with pro-fibrotic changes in cardiac tissue, contributing to the progression of heart failure, the occurrence of MACEs, and a generally poor prognosis. We are currently in an era of large-scale transcriptomic and proteomic studies, which broadly and comprehensively analyse the dysregulated expression of genes and proteins in patients with HCM. These studies have identified hub genes associated with the pathogenesis of HCM, highlighting potential therapeutic targets. Several signalling pathways are implicated in cardiac fibrosis, with the TGF-β cascade being the most widely known and investigated. Current evidence supports the role of TGF-β in the fibrotic changes observed in HCM. However, further studies are needed to explore differences between animal and human findings, as upregulated isoforms appear to vary between species. Importantly, monitoring the levels or expression of pro-fibrotic pathways could improve our ability to estimate prognosis in patients with HCM.
